# Substitutive proprioception feedback of a prosthetic wrist by electrotactile stimulation

**DOI:** 10.3389/fnins.2023.1135687

**Published:** 2023-02-21

**Authors:** Yichen Han, Yinping Lu, Yufeng Zuo, Hongliang Song, Chih-Hong Chou, Xing Wang, Xiangxin Li, Lei Li, Chuanxin M. Niu, Wensheng Hou

**Affiliations:** ^1^Biomedical Engineering Department, Bioengineering College, Chongqing University, Chongqing, China; ^2^Laboratory of Neurorehabilitation Engineering, School of Biomedical Engineering, Shanghai Jiao Tong University, Shanghai, China; ^3^Shenzhen Institutes of Advanced Technology, Chinese Academy of Sciences (CAS), Shenzhen, Guangdong, China; ^4^Department of Rehabilitation, Southwest Hospital, Army Medical University, Chongqing, China; ^5^Department of Rehabilitation Medicine, Ruijin Hospital, School of Medicine, Shanghai Jiao Tong University, Shanghai, China

**Keywords:** proprioceptive feedback, prosthetic wrist, transradial amputee, sensory substitution, electrotactile stimulation

## Abstract

**Objective:**

Sensory feedback of upper-limb prostheses is widely desired and studied. As important components of proprioception, position, and movement feedback help users to control prostheses better. Among various feedback methods, electrotactile stimulation is a potential method for coding proprioceptive information of a prosthesis. This study was motivated by the need for proprioception information for a prosthetic wrist. The flexion-extension (FE) position and movement information of the prosthetic wrist are transmitted back to the human body through multichannel electrotactile stimulation.

**Approach:**

We developed an electrotactile scheme to encode the FE position and movement of the prosthetic wrist and designed an integrated experimental platform. A preliminary experiment on the sensory threshold and discomfort threshold was performed. Then, two proprioceptive feedback experiments were performed: a position sense experiment (Exp 1) and a movement sense experiment (Exp 2). Each experiment included a learning session and a test session. The success rate (SR) and discrimination reaction time (DRT) were analyzed to evaluate the recognition effect. The acceptance of the electrotactile scheme was evaluated by a questionnaire.

**Main results:**

Our results showed that the average position SRs of five able-bodied subjects, amputee 1, and amputee 2 were 83.78, 97.78, and 84.44%, respectively. The average movement SR, and the direction and range SR of wrist movement in five able-bodied subjects were 76.25, 96.67%, respectively. Amputee 1 and amputee 2 had movement SRs of 87.78 and 90.00% and direction and range SRs of 64.58 and 77.08%, respectively. The average DRT of five able-bodied subjects was less than 1.5 s and that of amputees was less than 3.5 s.

**Conclusion:**

The results indicate that after a short period of learning, the subjects can sense the position and movement of wrist FE. The proposed substitutive scheme has the potential for amputees to sense a prosthetic wrist, thus enhancing the human-machine interaction.

## 1. Introduction

Prostheses help amputees improve their quality of life (Burçak et al., 2021). The increasing degrees of freedom (DoF) and more exquisite structure of current prostheses importantly contribute to the dexterity of movement (Bo et al., 2019; George et al., 2019). Several studies have found that in addition to comfort, function, appearance, and durability, prosthesis users also desire sensory feedback in the upper-limb prosthesis (Farina and Amsüss, 2016; Markovic et al., 2018; Wijk et al., 2020). Therefore, transmitting information about grasping force (Dosen et al., 2017), hand aperture (Witteveen et al., 2014), fingertip pressure (Wu et al., 2020), temperature (Ueda and Ishii, 2017), etc., of the prosthesis has been widely studied.

Proprioception of the limbs and trunk is arisen by several proprioceptors (Proske and Gandevia, 2012), such as the muscle spindle and Golgi tendon organ. Proprioceptive and tactile feedback are indispensable for sensorimotor integration in daily activities of human, especially for control of dexterous movement. For amputees, the muscle spindle, tendon, tactile receptor, and afferent fibers in the muscle of the residual limb are damaged and cannot work normally (Kaya et al., 2018). So proprioceptive substitution helps amputee sense the prosthesis, improves the confidence (Schiefer et al., 2018), and controls the prosthesis better (Grushko et al., 2020; Guémann et al., 2022). The senses of limb position and movement are significant because they provide us with one aspect of our self-awareness (Chen et al., 2021). Position feedback ranked second in a recent survey on requirements for feedback of prostheses (Stephens-Fripp et al., 2018). Position and movement sense (also called kinesthetic sense) are the subtypes of proprioception (Gilman, 2002; Proske and Gandevia, 2012). Earlier research found better performance in a myoelectric prosthetic arm when introducing vibration feedback to provide the user with the position information of the prosthetic elbow (Mann and Reimers, 1970). In recent decades, D’Anna et al. (2019) transmitted the position and tactile sense of a prosthetic finger by means of an invasive method, which enabled participants to identify the size of the object better when grasping. To reduce mental effort and improve the grasping performance of prostheses, Gonzalez et al. (2012) implemented position feedback of the prosthetic thumb, pointer, and middle finger through auditory stimulation. Vargas et al. (2021a) chose vibration stimulation to convey the static position and movement of the prosthetic fingers; as a result, the control accuracy of the joint angle was improved. Marasco et al. (2018) endowed amputees with a kinesthetic perception of dexterous prosthetic hands. The recent studies above have demonstrated the effectiveness of position and movement feedback.

The prosthetic wrist is crucially important for upper-limb prostheses (Fan et al., 2022) since it greatly contributes to the mobility of the hand and reduces additional compensation movements of the upper limb (Kyberd et al., 2011). The prosthetic wrist has three DoFs: flexion-extension (FE), ulnar-radial deviations (UR), and supination-pronation (SP) (Omarkulov et al., 2016); of these, SP and FE are the most requested (Demofonti et al., 2022). Therefore, there have been several approaches to sensory feedback of the prosthetic wrist. After Erwin employed a three-node tactor array to provide feedback information about the FE angle of a virtual wrist, the movement control of the wrist *via* electromyography was improved (Erwin and Sup, 2015). Kayhan et al. (2018) also developed a retractable skin stretching tractor, which provided feedback on the position of the prosthetic wrist during three DoF movements. Zheng et al. (2022) analyzed the effectiveness of wrist position feedback by comparing three kinds of feedback methods and demonstrated the importance of position feedback to the control of arm prostheses. In the above studies, it is undoubted that an appropriate and concise feedback method helps to promote the control and embodiment of the prosthesis (Page et al., 2018; Tchimino et al., 2022).

Homology and somatotopy are the priority factors affecting the acceptability of prosthetic sensory feedback methods because they affect the training periods that patients require (Raspopovic et al., 2021) and acceptance of the feedback device (Makin et al., 2017; Lan et al., 2019). In the literature, there are a variety of feedback methods, including invasive electrical stimulation (Schiefer et al., 2016; Vu et al., 2022), skin stretching (Battaglia et al., 2019), vibration (Vargas et al., 2021b), mechanical pressure (Godfrey et al., 2016), audio (Gonzalez et al., 2012), and electrotactile stimulation (Franceschi et al., 2017; Chai et al., 2022). Although the sensations induced by electrotactile stimulation are not somatotopic, users can learn to interpret the feedback with a few days of training (Bensmaia et al., 2020). Moreover, the electrotactile substitution system is easier to embed into upper-limb prostheses (Svensson et al., 2017) due to its benefits, such as non-invasiveness, portability, and low power consumption (Cornman et al., 2017). Therefore, electrotactile sensory substitution is one of the most promising bridges for connecting intelligent prosthetic fingertips and upper-limb amputees’ brains (Chai et al., 2014).

The effect of electrotactile feedback depends on the parameters of electrical stimulation, including intensity (Alotaibi et al., 2022), frequency (Farina et al., 2021; Graczyk et al., 2022), pulse width (Yang et al., 2012), spatial distribution (Rafiei et al., 2014), and temporal distribution (Nataletti et al., 2020). For multi-DoF intelligent prosthetics, the stimulation of multiple channels is more suitable for spatiotemporal encoding than that of a single channel because continuous stimulation causes skin adaptation (Buma et al., 2007) and limits the interpretation of changes in stimulation (Nataletti et al., 2022). Four channels electrotactile feedback method was proved to be feasible in lower-limb prostheses. Yang et al. (2012) provided feedback on the angles of a prosthetic knee and pressures at three sites on the prosthetic foot for transfemoral amputees through four electrodes, and the results showed increased temporal gait symmetry and augmented confidence when walking with sensory feedback compared to the no-feedback condition. More channels were also proven feasible, such as a 16-channel feedback scheme for transmitting four kinds of information about the hand and wrist to amputees (Štrbac et al., 2016), and different multichannel schemes were compared by the target-reaching task results of thirteen able-bodied subjects (Garenfeld et al., 2020). However, amputation results in cutaneous sensitivity changes (Koc et al., 2008; Templeton et al., 2018), which affects the comfort and dynamic range of electrotactile stimulation (Kaczmarek et al., 1991). It is reasonable to expect that amputee’s ability to use electrotactile stimulation for sensing wrist FE position and movement of prostheses may be substantially different from able-bodied subject. Besides, the wrist FE sensation includes not only movement direction but also movement range.

Therefore, this study aims to explore whether amputees can receive proprioceptive feedback on the prosthetic wrist through electrotactile method combined with spatial encoding and multiple electrodes. We proposed a multiple channels electrotactile stimulation scheme to provide wrist FE proprioception. In addition to recruiting amputees, we also recruited able-bodied subjects for comparison and validation. We hypothesized that the amputee’s performance of position sense and movement sense of the prosthetic wrist was different from that of able-bodied subjects and the success rate of position sense was higher than that of movement sense. To answer this question, the present study designed three experiment and a stimulation platform to verify the feasibility of the scheme by amputees’ recognition results, DRT, and questionnaire responses.

## 2. Materials and methods

### 2.1. Subject recruitment

Two transradial amputees (amputee 1: a 55-year-old male with electric shock amputation in 1989, amputee 2: a 60-year-old male with explosion amputation in 1980) were recruited for this research. Five able-bodied subjects (1 male, 4 females, 20∼25 years old) were recruited. All subjects met the following requirements: (a) not taking drugs that affect hormones or neurotransmitters in the last 30 days, (b) no electromagnetic hypersensitivity, (c) no psychiatric or cognitive disorder, and (d) experience using a myoelectric prosthesis. The experimental procedure was approved by the Chongqing University Three Gorges Hospital Ethics Committee (2021-KY-24). All subjects signed informed consent forms before the experiments, which includes the stimulation and prompts they would receive and what operations they needed to perform in the experiment.

### 2.2. Experimental setup

**FIGURE 1:**
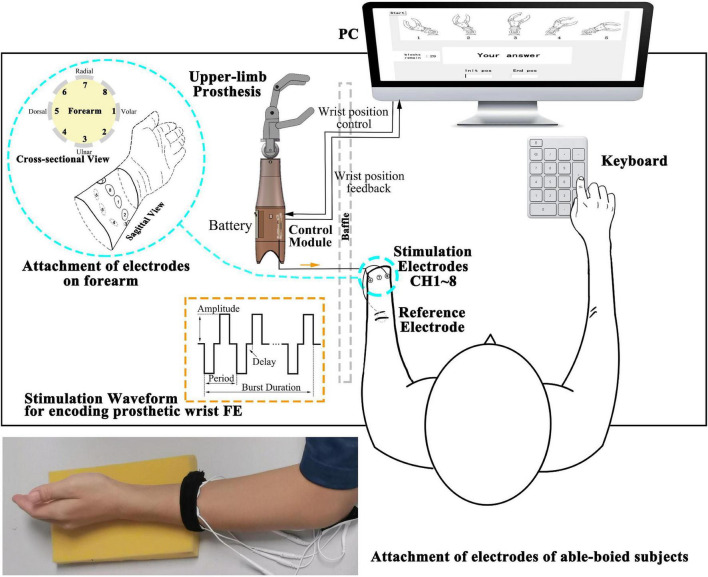
Illustration of the modules comprising the experimental platform. The control module and battery are embedded in the shell of the upper-limb prosthesis. The control module receives the wrist position control signal from the PC to drive the prosthesis and provided feedback on the wrist position to the PC. The square biphasic current stimulation waveforms in orange dashed line rectangle (amplitude, period, delay, and burst duration as adjustable parameters) are generated and conveyed to the subjects through electrodes. A cross-sectional and sagittal view of eight stimulation electrodes distribution around the forearm are shown in the enlarged blue circle. CH1 (Channel 1) is on the volar side, and eight channels are equally attached and arranged along the pronation direction. Subjects perceive the stimulation and input the answer by a keyboard. The photograph at the bottom shows the attachment of electrodes of an able-bodied subject.

The experimental platform mainly includes a PC, a control module, an upper-limb prosthesis and other devices, as shown in Figure 1. We independently designed, drew, and welded the control module and integrated the parts above to perform the following experiment.

(a) A host PC [Intel^®^ Core (TM) i7-7700HQ CPU at 2.80 GHz, 8 GB RAM] was used for running a Python program. A 22″ screen was used to provide guidance to the subjects, and the graphical user interface (GUI) created in the tkinter library was used for user input. The input content and discrimination reaction time (DRT) were saved in .csv format. The program called the pyserial library gives the prosthesis control signal and communicates with the microcontroller.
(b) The control module is used for outputting multiple channels electrotactile stimulation and driving the prosthesis, including main control board and daughter board. Main control board: (I) microcontroller minimum system (STM32F103RCT6) for generating PWM waveforms, selecting stimulation channels (CH1-CH8) and providing motor control signal, etc., (II) a chip for communicating with the PC, (III) interfaces that connect to the other device. Daughter board: (I) an H-bridge circuit, a constant current source, 70 V DC power supply, a quadruple high-current motor driver for executing prosthesis control signals, (II) solid-state relays as actuators for generating stimulation waveforms (square biphasic current waveforms). (III) Interfaces that connect to the motor of the prosthesis and other devices. The main control board and daughter board are connected by male and female headers (board to board). The shape of the boards is a rounded rectangle (60 mm*37 mm). The control module is connected to the inner shell of the prosthetic limb by screws.
(c) Upper limb prosthesis (SJQ21 SJS32 left hand, Danyang Prosthetic Factory, China) includes two DC micromotors (FAULHABER 2224006SR with magnetoelectric encoder IEH2-4096). The encoder feeds back the angle of the prosthetic wrist. This prosthesis supports two DoFs: hand aperture opening-closing and wrist FE. This strengthens the connection between the electrotactile scheme and the prosthesis. The inner shell of the prosthetic limb has screw holes for fixing the control module.
(d) Other devices: (I) Round hydrogel electrodes were used as the 1st to 8th stimulation channel (CH1∼CH8) and reference channel (Ref) (diameter = 2 cm and 5 cm, Shenzhen Baijianda Technology Development Co., Ltd., Shenzhen, China), (II) a 3.5″ TFT LCD was used to adjust the parameters of the stimulation waveform, and (III) a chargeable 9 V lithium battery was used as a power supply embedded in the prosthetic limb shell.

The parameters of the biphasic current waveform are adjustable (orange dashed rectangle in Figure 1): frequency (reciprocal of period) = 100∼500 Hz (100 Hz increments), pulse width = 100∼500 μs (100 μs increments), delay = 100∼500 μs (100 μs increments), current amplitude = 0∼8 mA (0.25 mA increments, 5 mA max for position and movement sense experiment), and burst duration = 0.5∼1 second (100 ms increments).

All subjects were required to sit on a chair in a comfortable posture; the able-bodied subjects’ dominant arms were placed on a sponge pad, and the plane of the palms was perpendicular to the ground. Amputees placed the residual limb on a sponge pad as well and were asked to keep the phantom palm in a straight (ST) position. For consistency, the circumference of 10–12 cm above the styloid process of the ulna and 2–4 cm above the amputation end were the places where able-bodied and amputees attached stimulation electrodes, respectively. A reference electrode was attached to the olecranon for each subject. CH1 is on the volar side, and eight channels were equally attached and arranged along the pronation direction. The connecting line of the centers of eight circular electrodes formed a plane perpendicular to the connecting line of the wrist and elbow (Figure 1).

### 2.3. Preliminary experiment of stimulation range selection

First, we conducted a preliminary experiment to explore the forearm skin sensory threshold and discomfort threshold of each subject. Referring to the general experimental paradigm of electrotactile evoked sensation (Chai et al., 2015; Zheng and Hu, 2018), we fixed the frequency of all electrical stimulation at 200 Hz, the pulse width at 500 μs, and the delay at 100 μs. Taking the ith channel as an example, the current amplitude was incremented from 0 μA in steps of 500 μA. Each stimulation lasted for 1 s and was then followed by a 10 s rest period. Once the stimulation was perceived, it was repeated 3 times to ensure that subjects perceived the stimulation clearly. The stimulation amplitude was recorded as the sensory threshold A_i1–up_ if perceived and then increased until the subjects felt discomfort, and the current amplitude was recorded as the discomfort threshold A_i2_. The current value was set to 40% of the maximal current, then it decreased in steps of 250 μA until the subjects could not perceive the stimulation. The last current value was recorded as A_i1–down_. The maximum value between A_i1–up_ and A_i1–down_ was considered the sensory threshold A_i1_. In addition, subjects were asked to describe the perceived sensations of stimulation, such as pressure, vibration, numbness, and pain, during this experiment. The current amplitude of each channel was fine-tuned by comparing the sensory threshold in neighboring channels to achieve similar tactile sensation across channels (Garenfeld et al., 2020).

### 2.4. Wrist FE static position sense experiment (Exp 1)

**FIGURE 2:**
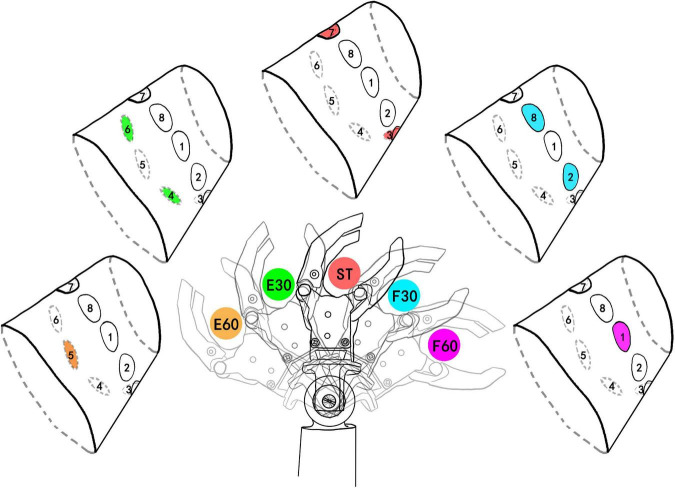
Five wrist FE (flexion and extension) positions and corresponding stimulating channels. The colored channel represents the active (under stimulating) channel. E60, extension 60°; E30, extension 30°; ST, straight 0°; F30, flexion 30°; F60, flexion 60°. E60 and F60 are limit positions of prosthetic wrist and they are coded by single channel. ST was defined as the initial position. The number after F and E was defined as the angle deviating from the ST position. The colors of active channels are consistent with the stimulation mode.

The study chose spatial coding to feedback the positions of wrist flexion and extension (FE), because spatial coding is easier for recognition than intensity coding or temporal coding. As shown in Figure 2, we primarily chose five angular positions of wrist FE with 30° of resolution, which were named extension 60° (E60), extension 30° (E30), ST, flexion 30° (F30), and flexion 60° (F60). As shown as attachment of electrodes in Figure 1, active electrodes among eight electrodes around the forearm correspond to the five positions of wrist FE. The position of active channel corresponds to the direction of wrist FE movement. As the limit positions of wrist FE movement, E60 and F60 are configured with single channel of active electrode. For example, the CH5 on the dorsal forearm corresponds to the limit extension direction of the prosthetic wrist, so it is coded as E60 position. The CH1 on the volar forearm corresponds to the limit flexion direction of the prosthetic wrist, so it is coded as F60 position. As the initial position of wrist FE movement, ST is configured with dual channels of active electrodes. The dual channels are related to CH7 and CH3. As the non-limited positions of wrist FE movement, E30 and F30 are configured with dual channels of active electrodes. For example, the CH 6 and CH4 close to the dorsal forearm corresponds to the extension position of the prosthetic wrist, so it is coded as E30 position. The CH 8 and CH2 close to the volar forearm corresponds to the flexion of the prosthetic wrist, so it is coded as F30 position. The recognition of a single channel is easier than that of dual channel (Geng et al., 2016), so we related the single channel to the limit position of wrist FE, informing the subject that the prosthetic wrist has moved to the limit position. The burst duration for each stimulation mode is fixed at 0.5 s. Before the electrical stimulation was executed, the prosthetic wrist moved to the corresponding position.

**FIGURE 3:**
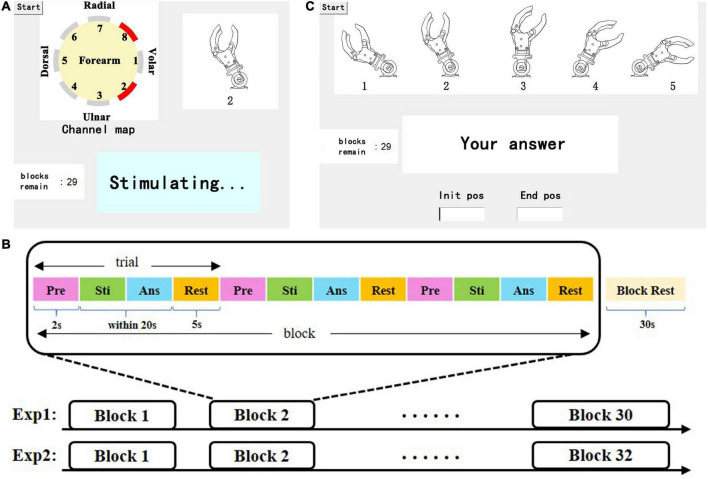
**(A)** Representation of the learning session, including a dynamic stimulating channel map, in which the red channel represents the active (under stimulating) channel, the illustrations of wrist FE, real-time status display, and the tip of the remaining blocks. **(B)** Depiction of the test session, including an illustration of the wrist position index (a static picture), a dialog box, and the tip of the remaining blocks. **(C)** Paradigm of the experiment, which consists of a certain number of blocks that consist of 3 of the same trials and 30 s of rest. Each trial consisted of four parts: preparation (Pre), stimulation (Sti), answer (Ans) and rest.

This experiment was composed of two sessions: a learning session and a test session. A learning session was arranged before the test session to familiarize the subjects with the electrotactile scheme. The stimulation modes occurred randomly. At the same time, the stimulating channel map and the corresponding prosthetic wrist state were displayed on the screen (Figure 3A). After 0.5 s of stimulation, there was a 10-s rest period. The learning session lasted approximately 10 min. After subjects passed an evaluation of learning, the test session would be executed.

The paradigm of the experiment in the test session is shown in Figure 3B. Each session contains 30 blocks (5 kinds of wrist FE positions *6 blocks), and each wrist FE position accounts for 6 blocks. Each block consisted of three identical trials. Each trial consisted of 2 s of preparation time during which a beep sound was played to prompt the subjects, a 0.5 s stimulation period, time for the participant to answer, and 5 s of rest time. Then, 30 s of rest was used to relieve mental fatigue. There was no reminder (including audition) in the test session. When waiting for the answer of subjects, a dialog box popped up on the screen. The subject needed to give the index of the prosthetic wrist FE position corresponding to the stimulation in the dialog box as fast as possible by pressing a single number and the enter key on the keyboard. The DRT was counted from the end of the stimulation to the time when the enter key was pressed. If there was no answer within 20 s, the trial was considered to be a failed recognition.

### 2.5. Wrist FE movement sense experiment (Exp 2)

**FIGURE 4:**
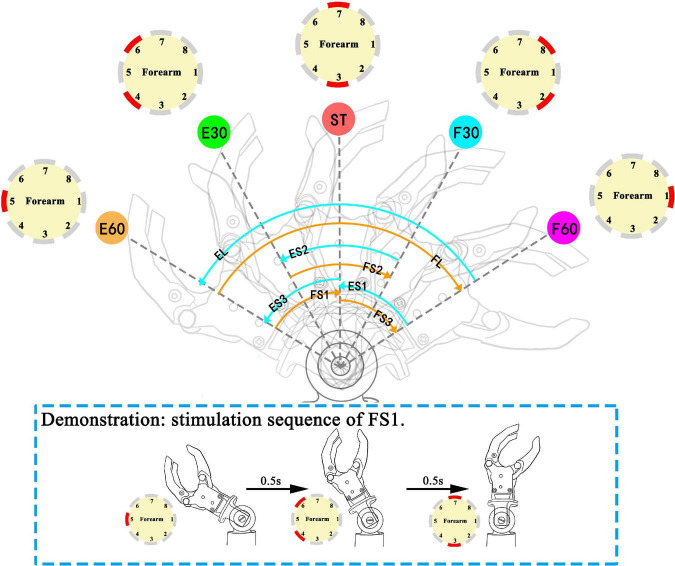
Movement modes in the movement experiment: ES (ES1, extension small 1; ES2, extension small 2; ES3, extension small 3), EL (extension large), FS (FS1, flexion small 1; FS2, flexion small 2; FS3, flexion small 3), and FL (flexion large). The sequence of active channels and the corresponding movement modes: ES1 (CH1→CH2, 8→CH3, 7), ES2 (CH2, 8→CH3, 7→CH4, 6), ES3 (CH3, 7→CH4, 6→CH5), EL (CH1→CH2, 8→CH3, 7→CH4, 6→CH5), FS1 (CH5→CH4, 6→CH3, 7), FS2 (CH4, 6→CH3, 7→CH2, 8), FS3 (CH3, 7→CH2, 8→CH1), FL (CH5→CH4, 6→CH3, 7→CH2, 8→CH1). The red channel in channel maps represents the active (under stimulating) channel. Demonstration for stimulation sequence of FS1 is illustrated in dashed line rectangle.

Based on the static position sense experiment, the study selected initial and end position from five wrist positions to form a movement mode. Our study chose eight movement modes from 20 combinations (5*4). Eight movement modes are: extension small 1 (ES1), extension small 2 (ES2), extension small 3 (ES3), extension large (EL), flexion small 1 (FS1), flexion small 2 (FS2), flexion small 3 (FS3), and flexion large (FL) (Figure 4). The variables include direction (F or E) and range (S:60° or L:120°). When the prosthetic wrist moved within the perception range of the five positions, the corresponding stimulation channels were active. After the prosthetic wrist moved in the next range of the preestablished positions, the previous channels were inactive. For consistency with the static position sense experiment, the burst duration on each electrode also lasted for 0.5 s. Therefore, the total duration of stimulation of ES1, 2, and 3 and FS1, 2, and 3 was 1.5 s, while that of EL and FL was 2.5 s.

The movement experiment also includes learning and test sessions. During the learning session, the subjects were provided with three kinds of guidance: 1. the movement of the prosthetic wrist, 2. the stimulating channel map, and 3. the dynamic illustrations of wrist FE movements on a 22″ screen. The above guidance helped subjects establish the connection between electrotactile stimulation and wrist movement to achieve a better learning effect. The learning session lasted approximately 15 min. The paradigm of the test session is similar to that of the static position sense experiment. The test session consisted of 32 blocks of random movement modes. To simplify user input, we numbered E60∼F60 as indexes 1∼5. Similarly, the subjects were asked to respond to the perceived movement by a keyboard. They needed to press the first index on the keyboard to represent the initial position, then the cursor was automatically switched to the next dialog box in which the second index represents the end position, and they finally pressed the enter key to submit the answer (Figure 3C). Similarly, we recorded the input and DRTs of the subjects. For consistency of the stimulation electrode position on the forearm in the two experiments, we used multiple reference positions such as the styloid process of ulna, olecranon, etc. In addition, we took photos of each subject’s forearm and marked the position of each electrodes with a color pen that is harmless to the skin.

Subjects’ subjective feelings need to be considered. To evaluate the acceptance of the electrotactile scheme in this study, after each subject completed the movement experiment, we distributed a questionnaire and invited the subjects to use a score from 1 to 5, where 5 represents the highest outcome, to rate the following aspects: 1. degree of pain and numbness, 2. the resolution of each channel, 3. the comfort of electrotactile stimulation, 4. intuitiveness, and 5. ease of learning.

### 2.6. Data and statistical analysis

We analyzed the sensory and discomfort thresholds and SR of each stimulation mode in two experiments. Specifically, in the movement experiment, we analyzed the SR from the following two aspects. (a) FE direction and range. Eight movements were divided into four categories (ES, FS, EL, and FL) by FE direction and range. For example, the given stimulation is ES2, while the answer is ES1, ES2, or ES3. This counts as a successful recognition in this aspect. (b) Each mode: only when both the initial position and end position were correctly identified can it be counted as a successful recognition. The DRT of success and wrong recognition of each stimulation were analyzed. The non-parametric Kolmogorov-Smirnov test (K-S test) and Bonferroni test were performed to detect the difference in each channel of sensory threshold and discomfort threshold and DRT, since previous tests have shown that all statistics failed to pass homogeneity of variance. The threshold for statistical significance was set at *p* < 0.05. Statistical analysis and graphing were performed in Prism 8.0.2 (GraphPad Software Inc, CA, USA).

## 3. Results

### 3.1. Preliminary experiment of stimulation range selection

#### 3.1.1. Individual electrotactile sensitivity

**FIGURE 5:**
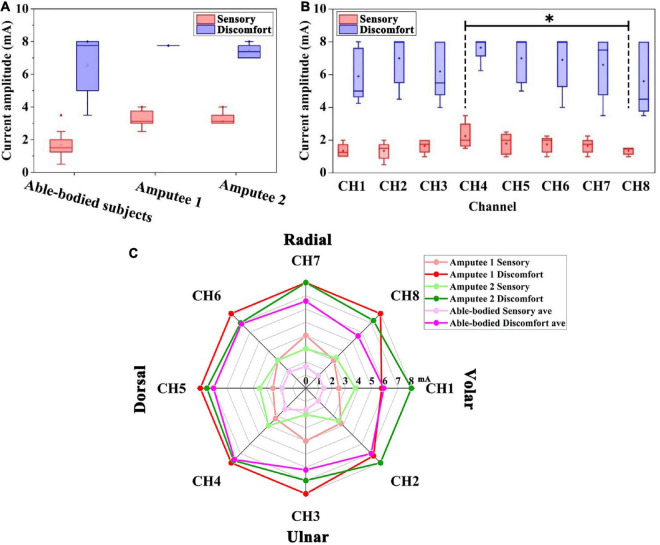
**(A)** Distribution of the sensory threshold and discomfort threshold of able-bodied subjects and two amputees. **(B)** Sensory threshold and discomfort threshold of able-bodied subjects among the eight positions. Black stars show the comparison of the sensory threshold for the eight positions. *p < 0.05. **(C)** Radar chart of the sensory threshold (light red, light green, and light purple) and discomfort threshold (red, green, and purple) around the forearm of five able-bodied subjects, amputee 1 and amputee 2. The dots in radar chart correspond to the threshold for eight electrodes around the forearm of the subjects.

The preliminary experiment examined the participants’ sensitivity to electrotactile stimulation. We analyzed the sensory and discomfort thresholds of able-bodied subjects and amputees, as shown in Figure 5A. Statistical analysis showed that the sensory thresholds of the 2 amputees (3.22 ± 0.57 mA, 3.25 ± 0.61 mA) were higher than those of the able-bodied subjects (1.64 ± 0.56 mA), but no such phenomenon was found in the discomfort thresholds. In addition, there was no significant difference between the sensory thresholds of the two amputees.

#### 3.1.2. Sensory sensitivity of different locations

The sensory thresholds at eight channels on the forearm of five able-bodied subjects are shown in Figure 5B. The sensory thresholds of the dorsal forearm and volar forearm sides are significantly different, which can be found in CH4 and CH8 (2.25 ± 0.79 mA and 1.35 ± 0.22 mA, *p* < 0.05, see the black stars in Figure 5B). Among all channels, CH1 and CH8 (both 1.35 mA) had the lowest mean values of sensory thresholds, and CH4 and CH5 (2.25 mA and 1.80 mA) had the highest sensory thresholds. The overall variability in the sensory threshold (1.64 ± 0.56 mA) was less than that in the discomfort threshold (6.61 ± 1.64 mA).

Figure 5C shows the distribution of thresholds around the forearm of all subjects. The sensory thresholds of each position of able-bodied subjects were lower than those of two amputees. The discomfort threshold of the CH1 channel of the amputee 1 showed an abnormal value of 5.75 mA, which was lower than that of able-bodied subjects (5.90 mA).

### 3.2. Proprioceptive feedback experiment

#### 3.2.1. Evaluation of wrist FE position sense

**FIGURE 6:**
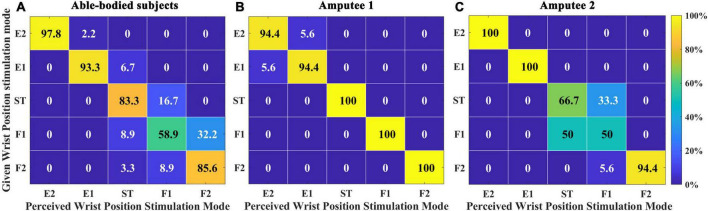
Confusion matrix quantifying the percentage of instances when comparing the perceived wrist position stimulation mode with a given stimulation mode in Exp 1. **(A)** Five able-bodied subjects, **(B)** amputee 1, and **(C)** amputee 2. The number in the each rectangle represents the SR (success rate), and the shade of the color represents the level of SR.

This experiment examined the subjects’ mastery of the position sense after a short period of study. Figure 6A represents the position recognition of five able-bodied subjects. The overall SR was 83.78 ± 3.69%. Able-bodied subjects had the highest SR for E60, which reached 97.78%. The SR for F30 was the lowest, only reaching 58.89%. Among the errors, 32.22% of F30 were identified as F60, and 8.82% of F60 were identified as F30. A total of 16.67% of those in the ST position were identified as F30. Figures 6B, C represent the SRs of amputee 1 and amputee 2, respectively. The total SR of amputee 1 was 97.78%. The SRs of the ST position, F30 and F60 reached 100%. The total SR of subject 2 was 82.22%. The SRs of E30 and E60 reached 100%, but the SR of F30 was only 50%. The subjects’ DRT was also an important index to evaluate the mastery of the electrotactile scheme.

**FIGURE 7:**
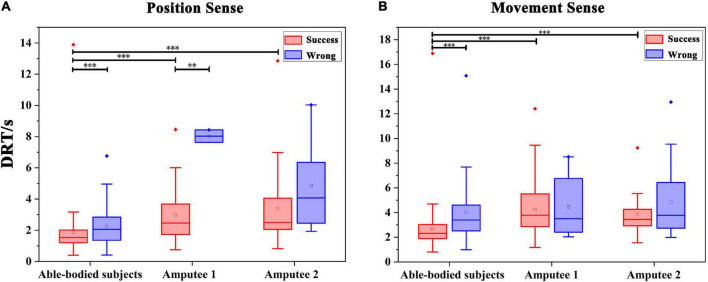
Statistics of the DRT (discrimination reaction time) in Exp 1 and Exp 2. **(A)** DRT for the evaluation of wrist FE position sense by all subjects. ^**^p < 0.01, ^***^p < 0.001. **(B)** DRT for all subjects spent in evaluating the wrist FE movement sense. ^***^p < 0.001.

The DRTs for successful recognition by able-bodied subjects, amputee 1 and amputee 2 were 1.891 ± 1.369 s, 2.974 ± 1.715 s, and 3.384 ± 2.342 s, respectively (Figure 7A). The DRTs for wrong recognition were 2.253 ± 1.287 s, 8.030 ± 0.568 s, and 4.861 ± 2.861 s, respectively. As shown in Figure 6, significant differences were observed between the DRT for successful and wrong recognition by five able-bodied subjects (*p* < 0.001) as well as that by amputee 1 (*p* < 0.01). The DRT for successful recognition by able-bodied subjects was significantly shorter than that of amputees (*p* < 0.001).

#### 3.2.2. Evaluation of wrist FE movement sense

**FIGURE 8:**
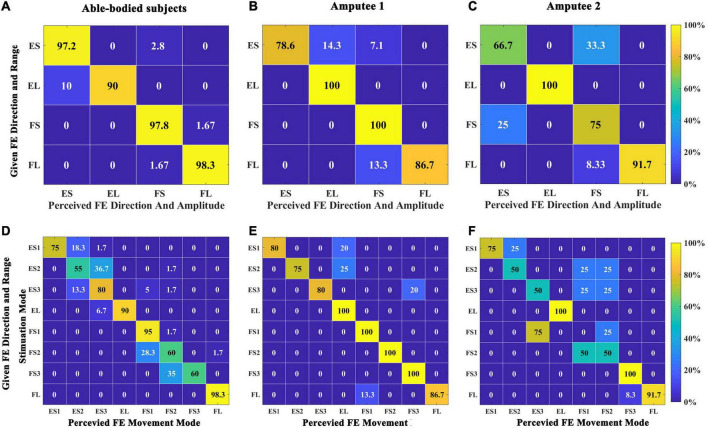
Confusion matrix quantifying the percentage of instances when comparing the perceived direction and range of wrist FE movements with the given stimulation in Exp 2. **(A)** Five able-bodied subjects. **(B)** Amputee 1. **(C)** Amputee 2. Confusion matrix quantifying the percentage of instances when comparing the perceived wrist movement stimulation mode with a given stimulation mode in Exp 2. **(D)** Five able-bodied subjects. **(E)** Amputee 1. **(F)** Amputee 2. The number in each rectangle represents the SR of the answer.

First, we calculated the SR of the direction and range of wrist FE movements. The total SR of able-bodied subjects was 96.67 ± 4.87%, in which the four categories were all over 90%, as shown in Figure 8A. The recognition effect of EL was slightly worse, and 10% of EL were recognized as ES. The total SR of amputee 1 was 90.00%. ES and FL were both 100%, and ES (83.3%) was the lowest, as shown in Figure 8B. The total SR of amputee 2 was 77.08%. ES (66.67%) had the worst SR, and 1/3 of that was recognized as FS. One-fourth of FS was recognized as ES, as shown in Figure 8C.

Figure 8D shows the recognition of stimulation modes for five able-bodied subjects, and the overall SR was 76.25 ± 18.97%. The SRs of EL, FS1, and FL were over 90%. The SR of FL was the highest, reaching 98%. Wrong recognitions mainly occurred around diagonal elements. ES2 had the lowest SR at 55%, with 37% wrongly answered as ES3. Figure 8E shows the SR of amputee 1. The total SR was 90.25%. EL, FS1, FS2, and FS3 all had SRs of 100%. The lowest SR was for ES2 (75%), and all wrong recognitions of ES2 were identified as E4. Figure 8F shows the SR of amputee 2. The total SR was 64.625%; the SRs for EL, FS3, and FL were higher than 95%, but those of ES2, ES3, and FS2 were 50%. None of the FS1 positions were identified, 75% of FS1 positions were wrongly identified as ES3, and the rest were identified as FS2.

The DRTs for successful recognition by able-bodied subjects, amputee 1 and amputee 2 were 2.666 ± 1.515 s, 4.238 ± 2.041 s, and 3.869 ± 1.528 s, respectively. The times for wrong recognitions were 4.207 ± 2.312 s, 4.459 ± 2.440 s, and 4.860 ± 2.771 s, respectively. As shown in Figure 7B, significant differences were observed between the DRT for successful and wrong recognition by five able-bodied subjects (*p* < 0.001). The DRT for successful recognition by able-bodied subjects was significantly shorter than that of amputees (*p* < 0.001). The results of the questionnaire are shown in Table 1 below.

**TABLE 1:** Results of the questionnaire.

Question	Able-bodied average	Amputee 1	Amputee 2
Pain, numbness	1.6	1	1
Resolution	3.6	4	5
Comfort	4.2	4	5
Intuitiveness	4.2	5	5
Ease	4.2	5	5

## 4. Discussion

We attempted to convey the wrist FE sense of a prosthesis by using electrotactile feedback to the amputee. Accordingly, we built an integrated platform and designed a spatiotemporal electrotactile scheme for mapping a group of prosthetic wrist position states. The study tested the recognition of position sense and movement sense in five able-bodied subjects and two amputees. The results showed that the coding could be well-recognized (average SR > 80%). This kind of coding is a potential method for proprioceptive sense feedback of prosthetic wrist FE in transradial amputees.

### 4.1. Preliminary experiment of stimulation range selection

In the preliminary experiment, the sensitivity of eight electrode channels was measured in all subjects. Differences in sensory thresholds between volar forearms and dorsal forearms of able-bodied subjects (Figure 5) may be due to anatomical structures. The average sensory thresholds on the volar side (such as for CH1, CH2, and CH8) of able-bodied subjects were lower than those on the dorsal side (CH4 and CH5). Transcutaneous electrical stimulation can not only act on skin to induce superficial sensation but also activate afferent sensory nerves. If the nerve distribution in this area is more superficial, the threshold is lower. The muscle spindle in the middle of the muscle is in a relatively superficial position under the skin, which results in the lowest mean sensory threshold of its sensory nerve. The muscles at the CH4 position are relatively thick, and the muscle spindles in the middle of the muscles are deeply distributed under the skin, resulting in a higher sensory threshold. Stimulation close to the median nerves may induce uncomfortable numbness. Therefore, we tried to avoid this volar forearm area or reduce the amplitude in these channels.

The difference in electrotactile sensitivity between the amputees and the able-bodied subjects was the sensory threshold (Figures 5A, C). The sensory thresholds in the two amputees were higher than those of the able-bodied subjects. The higher sensory threshold indicates that the nervous system of amputees needs to be injected with more stimulation energy to produce a similar sensory type to that of healthy subjects. The results reflected that amputee’s sensitivity is decreased than healthy subjects. This may be due to the sensory nerve impairment caused by transradial amputation. Kosasih and Silver-Thorn (1998) showed that unilateral tibial amputation caused superficial pain, vibration, and/or impaired touch sensation. In addition, electrotactile sensation is also relevant to mechanoreceptors distribution (He et al., 2016), reason and time of amputation, the age of subjects, etc. The discomfort threshold of CH1 was rather low in amputee 1. This might have been caused by the amputation operation performed on this subject. The skin sensitivity of the residual limb varies widely in space. Therefore, it is necessary to set the amplitude current independently on each channel.

The real current amplitude in each subject was fine-tuned around the sensory threshold to induce similar tactile sensation. Considering that long-term stimulation can induce sensory adaptation and numbness, we chose a relatively small amplitude for each channel, but this may have decreased the channel discrimination.

### 4.2. Proprioceptive feedback experiment

The study employed five kinds of stimulation modes to code the FE position of the prosthetic wrist, and the study also employed eight kinds of stimulation modes to code the FE movement of the prosthetic wrist.

For position sense, the test results in all able-bodied subjects preliminarily demonstrated the effectiveness of the electrotactile scheme. Upon further analysis of the wrongly recognized positions, dual-channel stimulation mode (F30, ST, E30) often reduced the difference between two channels, such as in the F30, F60, and ST positions. In our limited results, the SR of amputees was not inferior to that of able-bodied subjects. The possible reason is that amputation leads to different neural plasticity outcomes between amputees and able-bodied subjects with intact proprioceptive circuits (Di Pino et al., 2009; Terlaak et al., 2015). The overall SR of amputee 1 was better than that of subject 2. The slightly higher learning ability of subject 1 is a possible reason for this result, and the influencing factors might include subject 1’s younger age, higher education level, and higher economic level. Subject 2 did not clearly distinguish between ST and F30 positions. The possible reason for this result is that since the upper arm is not an ideal cylinder, CH3 and CH7 are close to CH2 and CH8. The induced sensations may also be quite similar, making them difficult to distinguish. This wrong recognition caused by spatial adjacency was also found for able-bodied subjects.

For movement sense, a temporal combination of multiple position sensations, hence the subject needs to perceive not only spatial change but also temporal change (F direction or E direction). Therefore, we predicted that the success rate of movement sense is lower than that of position sense. The results are consistent with the prediction. All subjects had a high SR for both the direction and the range of movement. Amputee 2 acquired a better SR in large-range FE movements than in small-range movements. For the same speed of movement in the prosthetic wrist, the burst duration of a large-range movement is longer than that of a small-range movement. Therefore, subjects can easily distinguish the large-angle range and the small-angle range at the time of stimulation. From the results of each stimulation mode, ES2, FS2, and FS3 were poorly recognized by able-bodied subjects. We speculate that the reason for this outcome is that the three modes all included position-F30, which was poorly recognized by able-bodied subjects in the position sense experiment (58.89%). The SR of amputee 1 was not lower than that of the able-bodied subjects, which may be due to the subject’s better performance in position learning. Different sensory threshold and recognition results all indicated that there is difference between amputees and able-bodied subjects. Similar interesting phenomena have also been observed. Both the amplitude and latency of the maximum ERP peaks for the amputee were smaller than those for the able-bodied subjects (Wang et al., 2022). One possible explanation is that the peripheral nerves regenerated in the stump were different from the intact one in structure and characteristics. Perhaps the nerve fibers in the residual stump may be fewer, and less sensitive compared to those in the intact limb. Another possible reason may be that the sensory neural pathways in amputee are different. For the amputee, electrical stimulation directly actives nerve endings of stump, arouses sensations, and transfers to the brain. But for able-bodied subjects, the electrical signals are transmitted to the nerves in the hand and returned back to the brain. In addition, brain reorganization after amputation (Chai et al., 2015; Björkman et al., 2016) may lead to different process of the central nerve system. The SRs of ES1, ES2, and ES3 were slightly lower than those of the remaining movement modes, which can be explained by the slightly lower SR (94.44%) of position sense for F60 and F30 than that for the other positions (100%). Seventy-five percent of FS1 (E60→E30→ST) positions were wrongly recognized as ES3 (ST→E60→E30) by amputee 2; these positions consist of two completely opposite movement modes. Because the subject’s recognition of the ST condition in the position sense experiment was low, the subject did not recognize the end position. Poor recognition of ST and F30 conditions was found in position sense experiments, resulting in poor recognition of ES2, ES3, and FS2 movements. We believe that for this subject, the confusion of a single position in this small-angle range movement misled the perception of the entire movement. When encoding wrist FE movements, F30, ST, and E30 using dual-channel stimulation may lead to confusion in the subjects’ perception.

For movement recognition, the DRT of able-bodied subjects was shorter than that of the two amputees, likely for the same reason mentioned for the wrist FE position sense. The DRTs of the two amputees did not show obvious differences in either successful recognition or wrong recognition. Both subjects were possibly confident that they had mastered the coding after their learning session for position sense and movement sense. In the learning session, all subjects were asked to watch the moving prosthetic limb or the wrist diagram on the display when they perceived the electrotactile stimulation. Other studies have shown that visual and tactile sensory systems share common features in object recognition, which proved that these systems have the potential to promote each other in the process of learning and cognition (Gonzalez et al., 2012; Tabrik et al., 2021). For transradial amputees, proprioception interruption caused by a missing wrist and hand decreased activity in their sensorimotor cortex circuit. After amputees underwent the above multisensory substitution training, their perceptual learning activity was induced in their sensorimotor cortex (Proulx et al., 2014). In our experiment, intuition guided the subjects to integrate the designed code method and prosthetic wrist movement without causing too much of a learning burden. The subjective responses of the two amputees to intuitiveness and ease of learning were in line with our expectations. Sensory feedback is important in the rehabilitation process of amputees who lack limb sensation, and the application of this system to somatosensory and motor training is expected to lead to enhanced motor and sensory cortical activation. The subjective responses of the two amputees to intuitiveness and ease of learning were in line with our expectations. Sensory feedback is important in the rehabilitation process of amputees who lack limb sensation, and the application of this system to somatosensory and motor training is expected to lead to enhanced motor and sensory cortical activation.

The current research had some limitations. First, too few amputees lead to the lack of universality of results and we will increase the number of amputees in future. Second, more evaluations, such as the time stability of the coding in combination with the forgetting curve (D’Anna et al., 2019), control performance of a prosthetic hand (Luo et al., 2021), and the activation of sensory cortex and mental burden through electroencephalogram (EEG) and functional near-infrared spectroscopy (fNIRS) (Midha et al., 2021; Zhu et al., 2021), need to be performed and the results need to be verified. Third, we focused on more reasonable experimental paradigms by setting the stimulation time and rest time (Buma et al., 2007; Marion et al., 2013).

## 5. Conclusion

In conclusion, this study demonstrated that our multichannel electrotactile substitutive scheme can provide effective prosthetic wrist FE proprioception information. The experimental results of the position sense and movement sense of two transradial amputees and five able-bodied subjects showed that after a short period of learning, the subjects can quickly grasp the electrotactile scheme to clearly identify the position and movement of the prosthesis. After simple improvement, the platform can be used in upper-limb prostheses to provide wrist proprioception feedback to transradial amputees, thereby improving subjects’ acceptance of the prosthesis.

## Data availability statement

The original contributions presented in this study are included in this article/supplementary material, further inquiries can be directed to the corresponding author.

## Ethics statement

The studies involving human participants were reviewed and approved by Chongqing University Three Gorges Hospital Ethics Committee. The patients/participants provided their written informed consent to participate in this study.

## Author contributions

XW, YH, WH, C-HC, CN, and YL designed the experiments. YH and YL conducted the experiments, analyzed the results, and created the figures. XW, YH, and YZ wrote the manuscript. HS participated in picture drawing. LL recruited the participants and was responsible for all the clinical activities. C-HC, WH, CN, and XL modified the manuscript. All authors reviewed the manuscript and approved the submitted version.
